# Supplementary figures and images for: Study on the relationship between microbial composition within obstructive biliary stents and the severity of obstruction and duration of stent placement
Source: PLoS One. 2025 Jan 9;20(1):e0317230. doi: 10.1371/journal.pone.0317230 (PMC11717289; doi:10.1371/journal.pone.0317230)

S1 Fig

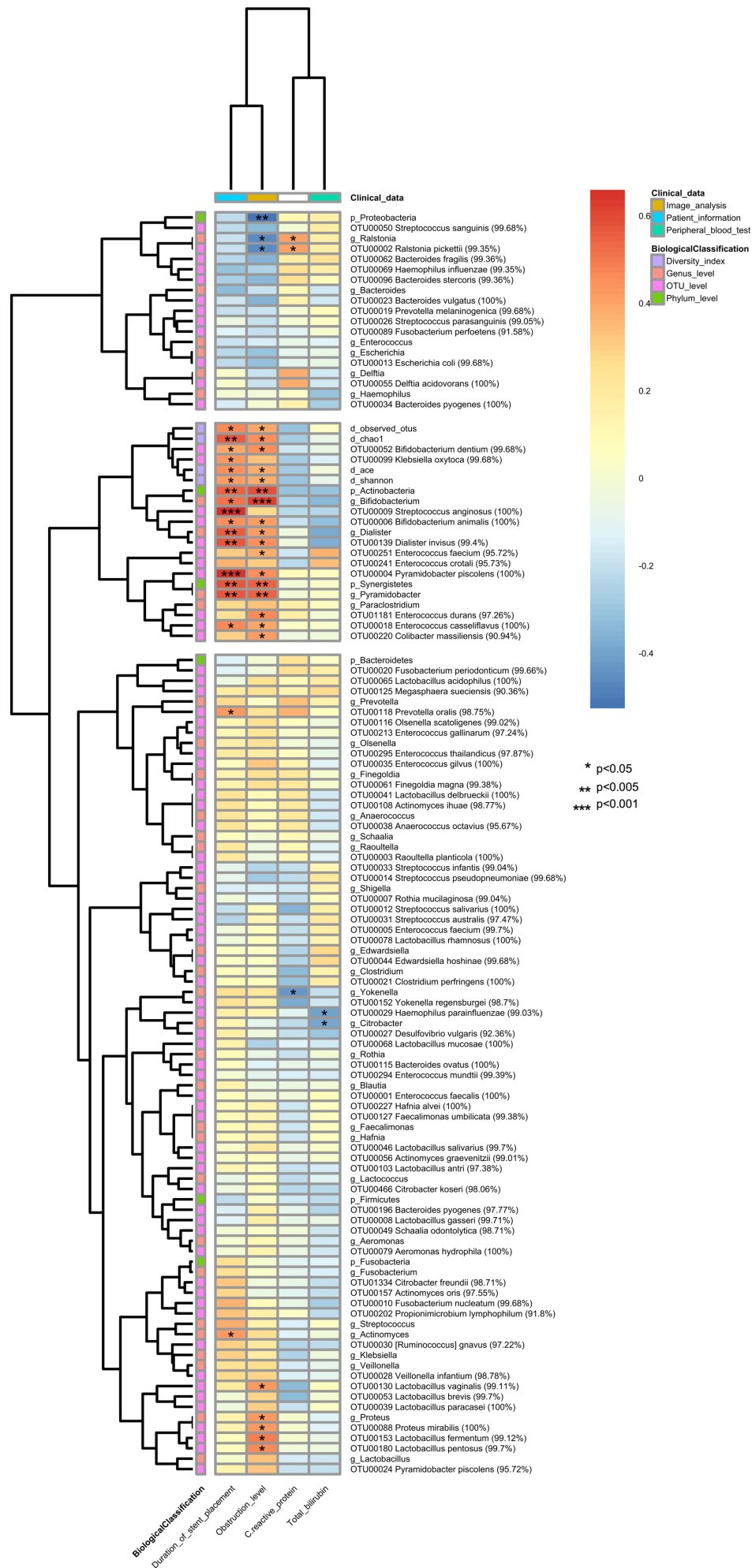

Supplement: S1 Fig — The black line in each scatter plot represents a linear regression line; the region in grey denotes 95% CIs. The color of each dot represents the disease that caused the biliary obstruction at the time of the initial endoscopic drainage. (PDF) [file pone.0317230.s002.pdf]
