# Supplementary material for: Study on the relationship between microbial composition within obstructive biliary stents and the severity of obstruction and duration of stent placement
Source: PLoS One. 2025 Jan 9;20(1):e0317230. doi: 10.1371/journal.pone.0317230 (PMC11717289; doi:10.1371/journal.pone.0317230)
Supplement: S1 Table — *Spearman’s correlation coefficient between each profile and severity of stent occlusion. Occlusion level calculated by image-analysis of biliary sludge inside the stent with Image J platform. OTU, operational taxonomic unit; ACE, abundance-based coverage estimator. (PDF) [file pone.0317230.s003.pdf]

S2 Table

The correlation between stent occlusion level (%) and the microbial profile

		Spearman's rho* (vs stent occlusion level [%])	p-value
Diversity indices	Chao1 index	0.45	0.02
	Observed OTUs	0.39	0.05
	ACE	0.38	0.05
	Shannon index	0.38	0.05
Abundance at the phylum level	Actinobacteria	0.59	0.001
	Synergistetes	0.54	0.003
	Proteobacteria	-0.53	0.005
Abundance at the genus level	Bifidobacterium	0.62	<0.001
	Pyramidobacter	0.54	0.003
	Proteus	0.43	0.03
	Dialister	0.42	0.03
	Ralstonia	-0.46	0.02
Abundance at OTU level	OTU00153 Lactobacillus fermentum (99.12%)	0.49	0.009
	OTU00180 Lactobacillus pentosus (99.7%)	0.47	0.01
	OTU01181 Enterococcus durans (97.26%)	0.46	0.02
	OTU00052 Bifidobacterium dentium (99.68%)	0.46	0.02
	OTU00139 Dialister invisus (99.4%)	0.45	0.02
	OTU00004 Pyramidobacter piscolens (100%)	0.45	0.02
	OTU00130 Lactobacillus vaginalis (99.11%)	0.43	0.02
	OTU00088 Proteus mirabilis (100%)	0.43	0.03
	OTU00006 Bifidobacterium animalis (100%)	0.42	0.03
	OTU00220 Colibacter massiliensis (90.94%)	0.42	0.03
	OTU00018 Enterococcus casseliflavus (100%)	0.40	0.04
	OTU00251 Enterococcus faecium (95.72%)	0.38	0.05
	OTU00002 Ralstonia pickettii (99.35%)	-0.46	0.02

*Spearman's correlation coefficient between each profile and severity of stent occlusion. Occlusion level calculated by image-analysis of biliary sludge inside the stent with Image J platform. OTU, operational taxonomic unit; ACE, abundance-based coverage estimator
