# Supplementary material for: Study on the relationship between microbial composition within obstructive biliary stents and the severity of obstruction and duration of stent placement
Source: PLoS One. 2025 Jan 9;20(1):e0317230. doi: 10.1371/journal.pone.0317230 (PMC11717289; doi:10.1371/journal.pone.0317230)
Supplement: S2 Table — *Spearman’s correlation coefficient between each profile and the duration of the stent placement. OTU, operational taxonomic unit; ACE, abundance-based coverage estimator. (PDF) [file pone.0317230.s004.pdf]

S3 Table

The correlation between the duration of the stent placement (days) and the microbial profile

		Spearman's rho* (vs duration of stent placement [days])	p-value
Diversity indices	Chao1	0.55	0.003
	Observed OTUs	0.46	0.02
	ACE	0.46	0.02
	Shannon index	0.42	0.03
Abundance at the phylum level	Synergistetes	0.57	0.002
	Actinobacteria	0.54	0.003
Abundance at the genus level	Pyramidobacter	0.57	0.002
	Dialister	0.56	0.003
	Bifidobacterium	0.51	0.006
	Actinomyces	0.42	0.03
Abundance at OTU level	OTU00009 Streptococcus anginosus (100%)	0.66	<0.001
	OTU00004 Pyramidobacter piscolens (100%)	0.64	<0.001
	OTU00139 Dialister invisus (99.4%)	0.57	0.002
	OTU00018 Enterococcus casseliflavus (100%)	0.46	0.02
	OTU00006 Bifidobacterium animalis (100%)	0.45	0.02
	OTU00099 Klebsiella oxytoca (99.68%)	0.43	0.02
	OTU00118 Prevotella oralis (98.75%)	0.43	0.03
	OTU00052 Bifidobacterium dentium (99.68%)	0.40	0.04

*Spearman's correlation coefficient between each profile and the duration of the stent placement. OTU, operational taxonomic unit; ACE, abundance-based coverage estimator
