# Supplementary material for: Study on the relationship between microbial composition within obstructive biliary stents and the severity of obstruction and duration of stent placement
Source: PLoS One. 2025 Jan 9;20(1):e0317230. doi: 10.1371/journal.pone.0317230 (PMC11717289; doi:10.1371/journal.pone.0317230)
Supplement: S3 Table — OTU, operational taxonomic unit; ACE, abundance-based coverage estimator. CI, confidence interval; SE, standard error. (PDF) [file pone.0317230.s005.pdf]

S4 Table

Correlation between stent occlusion level (%) and microbial profile, adjusted for the duration of stent placement using multi-regression analysis

		β	95% CI	SE	t	p-value
Diversity indices	Chao1 index	0.082	-0.16-0.33	0.12	0.70	0.49
	Observed OTUs	0.13	-0.34-0.59	0.22	0.57	0.58
	ACE	0.061	-0.16-0.28	0.10	0.59	0.57
	Shannon index	3.6	-10.2-17.4	6.5	0.55	0.59
Abundance at the phylum level	Actinobacteria	0.70	0.04-1.4	0.30	2.20	0.04
	Synergistetes	1.0	-0.4-2.5	0.69	1.50	0.15
	Proteobacteria	-0.28	-0.72-0.16	0.21	-1.40	0.19
Abundance at the genus level	Bifidobacterium	0.69	0.06-1.30	0.30	2.30	0.03
	Pyramidobacter	1.0	-0.4-2.5	0.7	1.50	0.16
	Proteus	3.9	-7.8-15.7	5.6	0.71	0.49
	Dialister	-7.4	-41.7-26.9	16.2	-0.46	0.65
	Ralstonia	-0.33	-1.40-0.78	0.53	-0.63	0.54
Abundance at OTU level	OTU00153 Lactobacillus fermentum (99.12%)	2.1	-2.0-6.2	1.9	1.10	0.28
	OTU00180 Lactobacillus pentosus (99.70%)	-2.2	-14.7-10.3	5.9	-0.38	0.71
	OTU01181 Enterococcus durans (97.26%)	-0.12	-1.30-1.08	0.57	-0.21	0.84
	OTU00052 Bifidobacterium dentium (99.68%)	-37.0	-1119.0-1045.0	512.7	-0.072	0.94
	OTU00139 Dialister invisus (99.40%)	-6.3	-40.7-28.1	16.3	-0.39	0.70
	OTU00004 Pyramidobacter piscolens (100%)	0.92	-0.94-2.80	0.88	1.10	0.31
	OTU00130 Lactobacillus vaginalis (99.11%)	7.4	-6.0-20.8	6.4	1.20	0.26
	OTU00088 Proteus mirabilis (100%)	4.1	-8.2-16.4	5.8	0.71	0.49
	OTU00006 Bifidobacterium animalis (100%)	0.64	-0.0027-1.29	0.31	2.10	0.05
	OTU00220 Colibacter massiliensis (90.94%)	64.1	15.5-112.6	23.0	2.80	0.01
	OTU00018 Enterococcus casseliflavus (100%)	1.08	-0.58-2.70	0.79	1.40	0.19
	OTU00251 Enterococcus faecium (95.72%)	-2.5	-20.1-15.2	8.4	-0.29	0.77
	OTU00002 Ralstonia pickettii (99.35%)	-0.33	-1.44-0.78	0.53	-0.63	0.54

*OTU, operational taxonomic unit; ACE, abundance-based coverage estimator; CI, confidence interval; SE, standard error
