# Supplementary material for: Study on the relationship between microbial composition within obstructive biliary stents and the severity of obstruction and duration of stent placement
Source: PLoS One. 2025 Jan 9;20(1):e0317230. doi: 10.1371/journal.pone.0317230 (PMC11717289; doi:10.1371/journal.pone.0317230)
Supplement: S4 Table — OTU, operational taxonomic unit; ACE, abundance-based coverage estimator. (PDF) [file pone.0317230.s006.pdf]

S5 Table

Differences between stent occlusion severity (%) and microbial profile according to the use of antibiotics (overall cases vs non-antibiotic cases)

		Overall cases n=27		Non-antibiotic cases n=20	
		Spearman's rho	p-value	Spearman's rho	p-value
Diversity indices	Chao1 index	0.45	0.02	0.42	0.07
	Observed OTUs	0.39	0.05	0.36	0.11
	ACE	0.38	0.05	0.40	0.08
	Shannon index	0.38	0.05	0.17	0.48
Abundance at the phylum level	Actinobacteria	0.59	0.001	0.55	0.01
	Synergistetes	0.54	0.003	0.44	0.05
	Proteobacteria	-0.53	0.005	-0.41	0.07
Abundance at the genus level	Bifidobacterium	0.62	<0.001	0.46	0.04
	Pyramidobacter	0.54	0.003	0.44	0.05
	Proteus	0.43	0.03	0.44	0.05
	Dialister	0.42	0.03	0.18	0.45
	Ralstonia	-0.46	0.02	-0.34	0.14
Abundance at OTU level	OTU00153 Lactobacillus fermentum (99.12%)	0.49	0.009	0.49	0.03
	OTU00180 Lactobacillus pentosus (99.7%)	0.47	0.01	0.33	0.16
	OTU01181 Enterococcus durans (97.26%)	0.46	0.02	0.34	0.15
	OTU00052 Bifidobacterium dentium (99.68%)	0.46	0.02	0.26	0.28
	OTU00139 Dialister invisus (99.4%)	0.45	0.02	0.25	0.30
	OTU00004 Pyramidobacter piscolens (100%)	0.45	0.02	0.32	0.17
	OTU00130 Lactobacillus vaginalis (99.11%)	0.43	0.02	0.43	0.06
	OTU00088 Proteus mirabilis (100%)	0.43	0.03	0.44	0.05
	OTU00006 Bifidobacterium animalis (100%)	0.42	0.03	0.36	0.12
	OTU00220 Colibacter massiliensis (90.94%)	0.42	0.03	0.25	0.30
	OTU00018 Enterococcus casseliflavus (100%)	0.40	0.04	0.18	0.44
	OTU00251 Enterococcus faecium (95.72%)	0.38	0.05	0.27	0.25
	OTU00002 Ralstonia pickettii (99.35%)	-0.46	0.02	-0.34	0.14

OTU, operational taxonomic unit; ACE, abundance-based coverage estimator
