# Supplementary material for: Study on the relationship between microbial composition within obstructive biliary stents and the severity of obstruction and duration of stent placement
Source: PLoS One. 2025 Jan 9;20(1):e0317230. doi: 10.1371/journal.pone.0317230 (PMC11717289; doi:10.1371/journal.pone.0317230)
Supplement: S5 Table — OTU, operational taxonomic unit; ACE, abundance-based coverage estimator. (PDF) [file pone.0317230.s007.pdf]

S6 Table

Differences between between duration of stent placement (days) and microbial profile according to the use of antibiotics (overall cases vs non-antibiotic cases)

		Overall cases n=27		Non-antibiotic cases n=20	
		Spearman's rho	p-value	Spearman's rho	p-value
Diversity indices	Chao1 index	0.55	0.003	0.39	0.09
	Observed OTUs	0.46	0.02	0.24	0.32
	ACE	0.46	0.02	0.32	0.17
	Shannon index	0.42	0.03	0.16	0.50
Abundance at the phylum level	Synergistetes	0.57	0.002	0.46	0.04
	Actinobacteria	0.54	0.003	0.34	0.14
Abundance at the genus level	Pyramidobacter	0.57	0.002	0.46	0.04
	Dialister	0.56	0.003	0.27	0.24
	Bifidobacterium	0.51	0.006	0.26	0.27
	Actinomyces	0.42	0.03	0.12	0.61
Abundance at OTU level	OTU00009 Streptococcus anginosus (100%)	0.66	<0.001	0.57	0.009
	OTU00004 Pyramidobacter piscolens (100%)	0.64	<0.001	0.61	0.004
	OTU00139 Dialister invisus (99.4%)	0.57	0.002	0.33	0.16
	OTU00018 Enterococcus casseliflavus (100%)	0.46	0.02	0.29	0.22
	OTU00006 Bifidobacterium animalis (100%)	0.45	0.02	0.40	0.08
	OTU00099 Klebsiella oxytoca (99.68%)	0.43	0.02	0.07	0.78
	OTU00118 Prevotella oralis (98.75%)	0.43	0.03	0.32	0.17
	OTU00052 Bifidobacterium dentium (99.68%)	0.40	0.04	0.04	0.87

OTU, operational taxonomic unit; ACE, abundance-based coverage estimator
